# Analysis of the prostate cancer cell line LNCaP transcriptome using a sequencing-by-synthesis approach

**DOI:** 10.1186/1471-2164-7-246

**Published:** 2006-09-29

**Authors:** Matthew N Bainbridge, René L Warren, Martin Hirst, Tammy Romanuik, Thomas Zeng, Anne Go, Allen Delaney, Malachi Griffith, Matthew Hickenbotham, Vincent Magrini, Elaine R Mardis, Marianne D Sadar, Asim S Siddiqui, Marco A Marra, Steven JM Jones

**Affiliations:** 1 British Columbia Cancer Agency (BCCA) Genome Sciences Centre, Vancouver, British Columbia, Canada; 2 Washington University School of Medicine, Genome Sequencing Center, St. Louis, Missouri 63108, USA

## Abstract

**Background:**

High throughput sequencing-by-synthesis is an emerging technology that allows the rapid production of millions of bases of data. Although the sequence reads are short, they can readily be used for re-sequencing. By re-sequencing the mRNA products of a cell, one may rapidly discover polymorphisms and splice variants particular to that cell.

**Results:**

We present the utility of massively parallel sequencing by synthesis for profiling the transcriptome of a human prostate cancer cell-line, LNCaP, that has been treated with the synthetic androgen, R1881. Through the generation of approximately 20 megabases (MB) of EST data, we detect transcription from over 10,000 gene loci, 25 previously undescribed alternative splicing events involving known exons, and over 1,500 high quality single nucleotide discrepancies with the reference human sequence. Further, we map nearly 10,000 ESTs to positions on the genome where no transcription is currently predicted to occur. We also characterize various obstacles with using sequencing by synthesis for transcriptome analysis and propose solutions to these problems.

**Conclusion:**

The use of high-throughput sequencing-by-synthesis methods for transcript profiling allows the specific and sensitive detection of many of a cell's transcripts, and also allows the discovery of high quality base discrepancies, and alternative splice variants. Thus, this technology may provide an effective means of understanding various disease states, discovering novel targets for disease treatment, and discovery of novel transcripts.

## Background

Large-scale characterization of mRNA populations has been approached through the generation of expressed sequence tags (ESTs), where single-pass sequencing reads are derived from cDNA clones [1,2]. This approach has proven to be extremely flexible in providing rapid identification of gene sequences, novel and alternatively spliced genes and for the annotation of genomic sequences [3]. Currently, over 30 million ESTs have been deposited into the public repository for expressed sequences, dbEST [4]. A drawback of this approach is the cost of generating sequencing reads, which, although continuing to decline, has dictated the ability for deep expression profiling of a given sample or tissue. Currently, the largest number of ESTs from a single tissue is 69,258, derived from pancreatic islet cells, and the median number for human EST datasets is 876. If a eukaryotic cell is estimated to contain approximately 3 × 10^5 ^mRNA molecules [5], then it is clear that deep sampling and quantization of specific mRNA populations is yet to be achieved through traditional EST sequencing. High sequencing costs combined with high sequence redundancy rates has led to normalization of cDNA libraries, though significantly improving the ability to derive novel transcript sequences, eliminates the utility of the EST data for any quantitative assessment of transcript abundance [6,7].

To address the use of mRNA sequencing to quantitatively assess transcript abundance the Serial Analysis of Gene Expression (SAGE) methodology was developed [5]. This approach provided restriction enzyme defined tags, initially fourteen base pairs in length, to be extracted from cDNA molecules. These are concatenated and subjected to single-pass sequencing, allowing a number of transcripts, typically 20–35 [8], to be identified in a single sequencing read. However, a few disadvantages remain with the technique: a number of transcripts lack the appropriate anchoring restriction tags to generate a SAGE tag and due to the relatively short sequences generated, usually 5–15% of tags will not map unambiguously to any gene locus [9]. As the SAGE tag is also expected to be derived from the 3' most anchoring restriction site, the ability of this technique to investigate transcript structure and splice variants is limited.

We report here on the application of a massively parallel sequencing approach utilizing sequencing-by-synthesis [10] as an efficient approach to generate ESTs. On genomic DNA, this approach has been shown to generate over 200,000 DNA sequences in a single machine run with an average read length of 110 base pairs [10], which is significantly shorter than those typically generated through Sanger-based capillary array electrophoresis sequencing. Using sequencing-by-synthesis random shotgun sequencing we hypothesize that these approaches will provide not only a quantitative measure of transcript abundance but also a survey of splice-variants within an mRNA population. As this approach does not require the cloning of the cDNA, it will also not be influenced by biases introduced by bacterial host-associated cloning bias.

We have chosen to perform experiments on the LNCaP human prostate cancer cell line [11] stimulated with synthetic androgen because it represents a well studied experimental resource and is a significant model for the study of prostate cancer.

## Results and discussion

A total of 181,279 ESTs were obtained which passed the default quality thresholds as determined by the manufacturer (454 Life Sciences Corporation, USA). A summary of the analysis of the ESTs is presented in Table 1. Initially, low quality bases were trimmed from the EST ends using trim2 [12] and the resulting sequences, with an average, minimum and maximum length of 102, 41, and 302 bp, respectively, were compared to the known and predicted human transcriptome [3]. 140,906 (77.7%) sequences matched directly by BLAST [13] to a specific human transcript with a p-value less than 9 × 10^-7^, while 40,373 (22.3%) of the sequences did not match with any known human transcribed sequence and hence potentially identify novel transcripts at this relatively high stringency. The 140,906 ESTs mapping to a known transcript cover 1.2 MB of the annotated human transcriptome and are available through dbEST [14].

**Figure 1:**
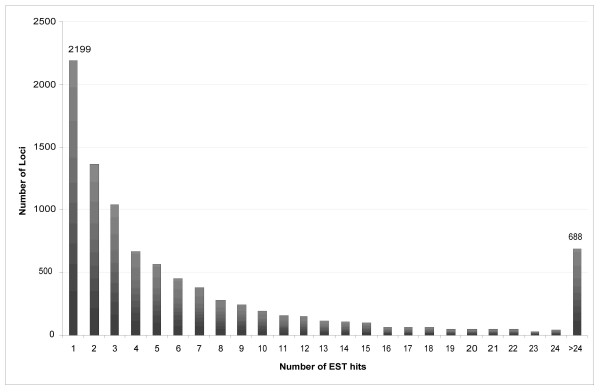
A histogram showing the number of gene loci hit by a given number of ESTs.

An histogram of the abundance of transcripts from annotated gene loci is shown in Figure 1. The ten most abundant transcripts are shown in Table 2. Further, we collected all genes detected in our library which are described by Ensembl as either being involved in cancer or expressed in the prostate and have made these available as a supplementary table [see Additional file 1]. Of the 9,173 loci for which transcription was detected (BLAST p ≤ 9 × 10^-7^) 2,199 were observed with a single EST sequence. Lowering the BLAST p-value to 0.05 allows 152,544 ESTs to map to 10,117 loci of which 2,417 have only one EST mapped to them (data not shown).

In order to determine whether our technique quantitatively measures transcript abundance, we compared our EST library to two SAGE libraries of R1881 treated LNCaP cells [15,16]. These two combined libraries have approximately 28,000 unique tags that we mapped unambiguously to 1,050 genes using DiscoverySpace [17].

Calculating the Pearson coefficient for all 9,173 genes gives a correlation value of 0.40. This value increases to 0.45 if we only consider genes that had at least one SAGE tag. Of our 10 most abundant genes, 3 (ENSG00000198899, 198886, 198763) are represented in the top 15 most abundant genes in the SAGE library. By reducing our stringency on which tags are deemed ambiguous we can successfully map an additional 3 genes of our top ten genes to the top 15 most abundant genes in the SAGE library.

**Figure 2:**
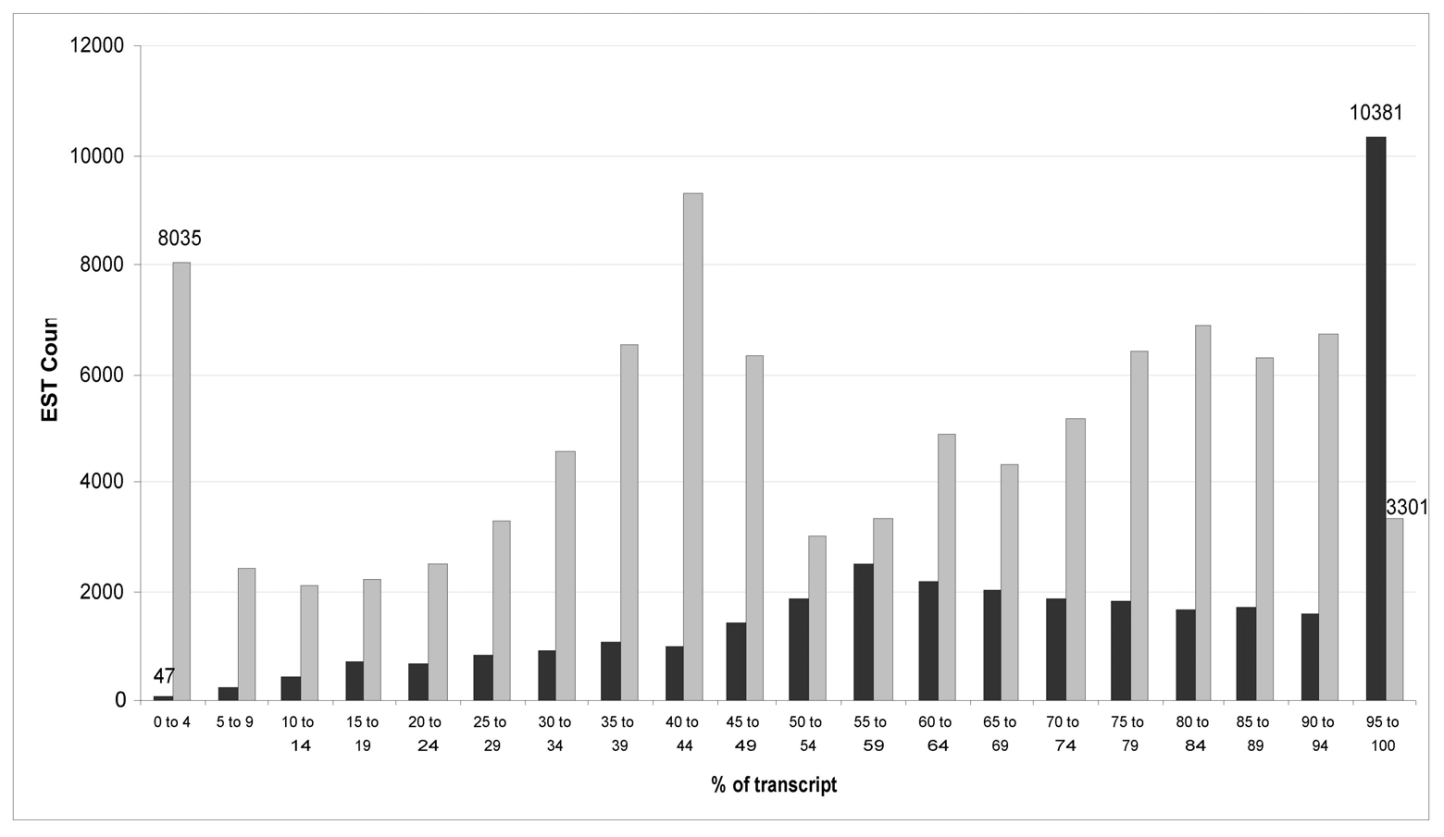
**A histogram showing the start of EST alignments to human transcript sequences (length > 500)**. Position is given as a percentage of the length of the transcript. ESTs which align to the positive or negative strands of the cDNA are shown in light or dark grey, respectively.

We studied the representation of the ESTs across known spliced transcripts (Figure 2). From this experiment, we observe four types of sequencing bias. The first favours the positive strand (coding) of the transcript. The second bias is seen at the 5' and 3' ends of the transcript. This occurs because the ends of a given transcript are readily available for sequencing, even if fragmentation of the cDNA is incomplete during the sample preparation (Methods). The increased representation of sequences in the mid-range of the transcript arises, almost entirely, to transcripts having lengths shorter than 1,200 bp. We found that such transcripts generally shear near the centre of the sequence (data not shown). Lastly, there is a general bias to the 3' end of the transcript that is likely due to incomplete cDNA synthesis across the entire length of the RNA transcript.

**Figure 3:**
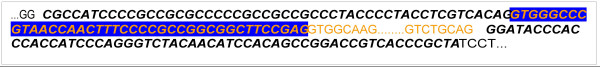
**Alternative splicing of Brain Protein I3 (ENSG00000164713) showing a short insertion between two exons**. 5' and 3' ends of two exons are shown in black text, interspaced by an intron (full sequence not shown) in orange. Base positions where the EST aligns to the transcript indicated with bold and italic type. The 40 base insertion is high-lighted in blue.

We investigated the ability of this approach to identify alternatively spliced transcripts within the mRNA population. By using BLAT [18] to map reads which showed good alignment to the transcriptome, but poor alignment at either end of the read, we discovered 25 (Table 3) previously unreported splice junctions that begin and end in a previously annotated exon and 106 novel alternative splice variants that map from a known exon and splice into intronic sequence. For all alternate splices, save 2, the event was demonstrated by a single EST. Figure 3 shows an alternative splicing event within the gene coding for Brain Protein I3 (*BRI3*) that causes a 40 base pair insertion between exons 1 and 2. This frame-shift occurs upstream of the transmembrane regions of the protein, as predicted by Ensembl [19], and although it does not cause a premature stop in the coding sequence, it eliminates the transmembrane domains of the protein as determined by InterProScan [20]. Interestingly, disruption of *BRI3 *has been implicated in tumor necrosis factor alpha (TNF-α) induced apoptosis resistance [21].

In areas where the base quality, as determined by the 454 sequencer, was exceptionally high, we utilized the EST data to detect high quality discrepancies (HQDs) in the LNCaP transcriptome. In this analysis, we required that discrepancies have a phred-like score >80 in order to be considered significant. This score threshold is set a such a level that we would require, at minimum, 3 sequences to confirm the presence of a HQD. Using this stringent approach we discovered 1,479 HQDs of which 86 (5.8%) were present in Ensembl's variations database [3], 29 showed variations at the same position but to a different base, and 1,364 were not described in Ensembl. For each HQD type, the mean and median base quality score were calculated (see Methods). The mean and median scores for confirmable HQDs are significantly higher (p ≤ 0.02) than for unconfirmable HDQs (Table 4). Although we do not dismiss all of the unconfirmable 1,393 HQDs as spurious, even under such an hypothesis, a significant (p ≤ 10^-45^) enrichment of characterized variations is found, as compared to random sampling.

We also used the ESTs for the discovery of unannontated genes. Of the 40,373 ESTs that did not map to the known human transcriptome at high stringency, 19,392 (48.0%) were successfully mapped to the entire human genome sequence at high confidence (p ≤ 5 × 10^-6^). Of these, 9,488 (48.9%) map mostly or entirely to an intron. The remaining 9,904 ESTs map intergenically, that is, to a region that is not known or predicted to contain an ORF. Further, 1,900 (19.2%) of these ESTs map to a region where there is no existing alignment feature in Ensembl (EST gene, or EST) and 380 (3.8%) align at least 20 Kbp away from a known gene.

**Figure 4:**
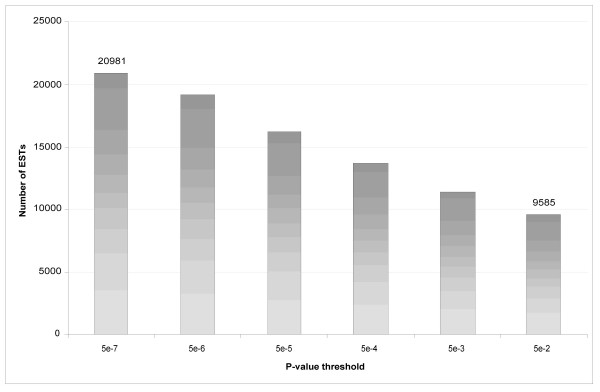
A histogram of ESTs that fail to map to the human genome at various p-values.

Of the 40,373 reads, 20,981 failed to map to the human genome at p ≤ 5 × 10^-6^. Figure 4 shows the distribution of ESTs that fail to map successfully to the human genome or transcriptome at various p-value thresholds. At p ≤ 0.05, 9,585 reads remain unmapped.

The 9,585 ESTs that failed to map to the human genome were then aligned to sequences in GenBank-nt (Dec 5^th^, 2005). 7,643 failed to map with a p-value ≤ 0.05, 605 reads mapped most strongly to human sequences and 587 reads were mapped to other organisms (Table 5). The 605 human sequences that failed to map to the human genome/transcriptome were missed for 2 reasons. First, because of the different nucleotide frequencies in the two databases, ESTs that have low complexity, and therefore high p-values, when mapped to the human genome (or transcriptome) have lower p-values when mapped to GenBank-nt. Second, the GenBank-nt database contained ESTs with splicing patterns not represented in the current Ensembl transcript annotation. Therefore, the ESTs that map to exon-exon junctions in these speculated genes will also have poor p-values. The 587 reads which were mapped to other organisms are a product of contamination of the sample. Although the contamination could have occurred before cDNA production, the nature of the contaminants suggests that this most likely occurred in the 454 nebulization process. The 7,643 remaining ESTs are almost certainly the product of poor quality sequencing. Although their quality values are not significantly different from the other reads, they are markedly shorter (average length of ~86 nt) which reduces the minimum possible p-value for these sequences.

## Conclusion

The data reported here show that massively parallel sequencing-by-synthesis methods can be used to successfully survey a transcriptome. Of the top 10 most abundant transcripts, 7 are involved with energy production and are located on the mitochondrial genome (Table 2). The over representation of metabolic genes may be indicative of the high energy requirements of the cancerous cell. Interestingly, 8 of the 25 novel splicing events listed in Table 3 also occur in genes directly involved with mitochondria and/or energy production, whereas 4 others are involved in translation or transcription and may have multiple effects on the cell. Further, we were able to identify the expression of over 10,117 different genes (p ≤ 0.05). Of these genes, approximately one-third are detected by only one or two ESTs (Figure 1), showing a low level of redundancy in the library and indicating that further sequence sampling likely would determine the transcription of significantly more gene loci.

This compares favourably to Affymetrix microarray experiments done with LNCaP, which typically find between two and eight thousand genes (Gene Expression Omibus (GEO); [22,23]; January 16^th^, 2006). A specific study of LNCaP cell expression was carried out by Oudes et al. [24] using both the Affymetrix profiling platform [25] and Massively Parallel Signature sequencing (MPSS) [26]. In this analysis, 9,841 genes were identified as being expressed using the Affymetrix technology (p ≤ 0.04) and 7,863 using MPSS. In total, Oudes et al. identified 9,841 genes expressed in LNCaP. Our approach compares favourably to MPSS, finding 16.6% more genes. Although we find 668 fewer genes than the Affymetrix approach at high stringency, using a lower BLAST alignment stringency (p ≤ 0.05), we discover 276 more genes than by the microarray-based approach.

We were able to identify 25 novel alternative exon splicing events from 20 MB of data in a stringent, high-throughput manner. We also discovered over four thousand ESTs that are entirely or partially intronic. These may originate from unprocessed mRNA or may represent novel or extended exons. Although it is not possible to fully determine the exact sequence of any of these interesting transcripts from 454 reads, this technique does identify transcripts which could be PCR-amplified and sequenced in their entirety.

Of the ~9,000 detected genes 76 are directly described as being involved with cancer or the prostate. The most highly expressed prostate cancer gene is Prostate Specific Androgen (PSA) and the most highly expressed cancer specific gene is Mindin. Both genes have been previously identified as strong prostate cancer markers [27,28] and both are in the top 40 most abundant genes in our EST library (data not shown). This would seem to indicate that our approach is capable of identifying genes important to prostate cancer pathology.

With respect to using 454 sequencing to measure transcript abundance, our results correlate modestly to those of SAGE, having Pearson coefficients between 0.4 and 0.45. However, these values are not significantly lower than correlations between long and short SAGE or SAGE and Affymetrix chips which generally lie between 0.4 and 0.65 [29]. The reason for our low correlation coefficients is likely due to a combination of factors. Most notably is that the long reads produced by 454 allow more tags to be mapped unambiguously to a gene as compared to SAGE where the short ESTs are much more likely to align to multiple loci. Further, the number of tags produced for a given transcript by 454 will depend on transcript length and shearing efficiency as well as transcript abundance. These latter factors make compensating for biases in transcript abundance difficult.

From the approximately 300,000 base pairs of EST sequence with a total phred-like score > 80, as assessed by the 454 base calling software, we determined approximately 1,500 high-quality discrepancies with respect to the human reference sequence (Table 4). This represents approximately one polymorphism per 200 base pairs. This rate is approximately 3–4 times higher than would be expected from the sequencing of DNA from a normal human diploid source [30]. This increased rate of polymorphism can possibly be attributed to the genomic instability and loss of DNA repair mechanisms that would have contributed to the original malignancy [31] as well as the number of passages the cell-line would have undergone since the original isolation in 1977, and during which additional mutations would have accrued [11].

Lastly, we were able to map 1,900 ESTs to regions in the human genome where there are neither genes nor other alignment features, such as ESTs (Table 1). This is consistent with studies using the Affymetrix technology which determined that 49% of transcribed bases determined on human chromosomes 21 and 22 fell outside regions containing a gene annotation [32].

This analysis also revealed the bias that occurs when sequencing short sequences of DNA by 454 sequencing (Figure 2). Due to poor or inconsistent nebulization of the cDNA sample, sequencing occurs more frequently at the 3' and 5' ends than at the middle of the DNA strand, and this gives uneven profiling of the underlying transcriptome. The 3' bias is compounded by incomplete (i.e. not full length) cDNA synthesis, which is known to bias the 3' ends of transcripts. Lastly, there is a bias to the coding strand of the transcript and the exact mechanism underlying this observation remains unclear. Fortunately, however, this last form of bias has little effect on the possibility of observing alternate splicing events or HDQs in a transcript. The former biases can likely be overcome by using an alternate method of fragmenting the cDNA such as random-hexamer primed PCR or possibly nebulizing to a smaller fragment size. We also discovered a minor difficulty with contaminating DNA in the sample preparation (Table 5). This highlights the sensitivity of 454 sequencing as well as the need to keep sample preparation clean and to be stringent when aligning sequence data to a target organism.

Much of the complexity in our analysis was due to the propensity of 454 sequencing to insert or delete bases in homopolymeric nucleotide runs [10]. This caused excessive penalties for gapping and other difficulties in the alignments when using a traditional alignment tool such as BLAST. Alternatively, BLAT tended to over-insert large gaps in the alignments because it suspected every insertion or deletion in the sequence to potentially be the start of an intron. Further use of this technology for transcriptional profiling would require the development of a tool, similar to BLAT, which does not greatly penalize gaps that begin in a homopolymeric region of the sequence and as a consequence, provides better prediction of intron-exon boundaries.

This work has shown that high-throughput sequencing using the 454 sequencing-by-synthesis approach is able to profile transcript abundance, and to discover nucleotide discrepancies and novel transcript splicing events.

## Methods

### Cell culture and mRNA preparation

LNCaP human prostate cancer cells (American Type Culture Collection^®^; Bethesda, MD) were maintained in RPMI-1640 media (StemCell Technologies; Vancouver, BC) supplemented with 10% fetal bovine serum (FBS; StemCell Technologies) and incubated at 37°C with 5% CO_2_. Cells at passage 38 were plated at a density of approximately 4 × 10^6 ^cells per T175 flask. Cells were serum-starved for 48 hours prior to treatment for 16 hours with 10 nM R1881 (PerkinElmer; Woodbridge, Canada). Cells were harvested and total RNA was extracted from the cells using TRIZOL^® ^Reagent (Invitrogen™ Life Technologies, Carlsbad, CA) following the manufacturer's instructions.

### cDNA preparation

RNA was assayed for quality and quantified using an Agilent 2100 Bioanalyzer (Agilent Technologies, Mississauga, ON) and RNA 6000 Nano LabChip kit (Caliper Technologies, Hopkinton, MA). Contaminating genomic DNA was removed from 1 mg of total RNA by DNAse1 treatment using DNAfree (Ambion, Austin, TX) following the manufacture's instructions. mRNA was isolated from total RNA using the MACS mRNA Kit (Miltenyi Biotec, Auburn, CA) following the manufacture's instructions with the exception of two additional washes prior to elution and a 3% final yield of mRNA (28 ug). cDNA was prepared from 12 ug of mRNA using the SuperScript Choice cDNA synthesis kit following the manufacture's instructions (Invitrogen Life Technologies, Carlsbad, CA). The resulting 7.1 ug of cDNA was concentrated to ~300 ng/ul by lyophilization prior to 454 sequencing.

### 454 sequencing

In preparation for 454 sequencing, the cDNA sample was nebulized to a mean fragment size of 600 ± 50 bp, end repaired and adapter ligated according to the standard procedures described previously [10]. After streptavidin bead enrichment and DNA denaturation, we recovered 5.21 E+10 single-stranded molecules/ul with an average size of 620 ± 50 bp that were titrated onto derivatized Sepharose beads and then amplified by emulsion PCR. A second streptavidin bead enrichment followed emulsion breaking, the bead-attached DNAs were denatured with NaOH, and sequencing primers were annealed. Two 454 sequencing runs were obtained from this library – the first on a 40 × 75 Picotitreplate™ (PTP) and the second on a 70 × 75 PTP. We followed standard post-run bioinformatics processing on the 454 platform to determine reads that passed various quality filters. These reads were used in our downstream analysis, as described.

### Sequence analysis

Sequences were first trimmed of low quality bases which can occur at the end of reads using trim2 (-M 10) [12]. The reads where then mapped to the human transcriptome (Ensembl cDNA, November 8^th ^2005) using wuBLAST [13] (version 2.0 May 10^th^, 2005) (-V100 -B100 -W25). BLAST hits with a *p*-value ≤ 9 × 10^-7^, which corresponds approximately to a 60 base pair contiguous perfect match in the data set, were considered to be successful hits against the transcriptome.

In order to determine that our mappings were real, we aligned with wuBLAST the lowest scoring hits (9 × 10^-8 ^≤ p < 9 × 10^-7^), 3,784 in total, against the GenBank-nt database. Of these 3,448 (91.1%) hit a human sequence most strongly. 191 (5.0%) hit a primate, usually with only 1 or 2 more matching bases than in the human alignment and the remaining 145 (3.8%) hit other organisms.

Of the reads that successfully hit the transcriptome those that were not aligned within 25 bp or more of the 3'- or 5'-end of the EST were considered gapped and were aligned against a collection of transcription units (Ensembl transcript, November 8^th^, 2005) using BLAT [18]. BLAT hits were considered better if BLAT extended the alignment of the EST by at least 25 bps and at least 25 bps were aligned on either side of any large gaps, if present, in the alignment (presumed to be intronic sequence). The BLAT hits were then evaluated on whether they over/under-ran a transcription unit, over/under-ran an exon, mapped from one exon to another, or mapped from an exon to an intron.

Sequences that did not map to the human transcriptome were then aligned with wuBLAST (-V100 -B100 -W15) to the human genome. Lower values of W (wordsize) were attempted but made little difference to the number of ESTs mapped (data not shown). The positions of significant hits (p ≤ 5 × 10^-6^), which correspond approximately to a 60 base pair contiguous perfect match in the data set, with respect to genes, introns, exons, ESTs and other DNA alignment features, were determined using the perl Ensembl API [33] (version 35) and Ensembl database (version 35). Reads that did not map against the human genome (p ≤ 0.05) were then aligned against the GenBank nucleotide database (Dec 5^th^, 2005) with wuBLAST.

### HQD analysis

High quality discrepancies (HQDs) were discovered by first using the alignments of ESTs to the transcriptome (described above). However, only very high quality alignments were kept, such that no alignment contained more than 3 mismatches, nor more than 9 gap positions. For every possible nucleotide X = (A or C or G or T), at every position Y, in a transcript, the number of ESTs that possessed base X at position Y in their alignment was calculated along with the combined phred-like [34,35] quality score for that base. The combined phred-like score is the sum of all phred scores from each EST that contributes to X at Y. A discrepancy was defined as any mismatch in an alignment. The discrepancy was considered high quality if the combined phred-like score for that base was >80. For example, suppose in a given transcript that we expect an 'A' at position 1000. If 4 ESTs align to this transcript over-top the 1000^th ^base, each with associated scores as follows: A (30), C (25), C (30), C(30), then at this position we have the canonical A with a score of 30, and a high quality discrepancy 'C' with a score of 85.

Each HQD was either confirmed by its presence in Ensembl's variance database, or marked as speculative due to its absence in Ensembl. The probability of observing 86 or more confirmable HQDs out of 1,479 total HQDs is given by the binomial distribution.

## Abbreviations

BLAST – Basic Local Alignment Search Tool

BLAT – Blast like alignment tool

EST – Expressed sequence tag

HDQ – High quality base discrepancy

MB – Megabase

SAGE – Serial analysis of gene expression

SNP – Single nucleotide polymorphism

## Authors' contributions

MB carried out primary analysis, wrote the software pipeline, and drafted the majority of the manuscript. RW assisted in software development, manuscript editing, and provided expertise in assemblies and alignments. Hirst, TZ, AG carried out culture growth, mRNA extraction, mRNA preparation, and cDNA generation. Hickenbotham, VM, EM carried out 454 sequencing, and provided expertise in interpreting 454 data and dealing with problems in the data. MG, AD provided expertise in alternative splice site discovery, SAGE-like analysis, and insights in the bias problems and aided extensively in editing the manuscript. TR, MS provided expertise in prostate cancer genomics and provided the cell culture. AS, MM provided expertise in data analysis and experimental design. SJ conceived, guided and helped interpret the experiment described herein, as well as assisted in drafting the manuscript. All authors have read and approved the manuscript.

## Supplementary Material

Additional File 1 **Prostate_and_Cancer_Genes**. A comma seperated values table, that contains genes which are discovered in the EST library and are described by Emsembl as being either cancer or proatste related. Click here for file

## Figures and Tables

**Table 1:** Summary analysis of EST to human transcriptome/genome mapping

Map Type	Count
**ESTs mapping to the human transcriptome (p ≤ 9 × 10^-7^)**	**140,906**
**ESTs mapping to the human genome and overlapping with a processed transcript**	**1261**
**ESTs which map to a transcribed region**	**8,221**
**ESTs which map to the human Genome alone**	**9,482**
**ESTs not correlating with the human genome (p ≤ 9 × 10^-5^)**	**20,981**
Total	181,279

**Table 2:** Top 10 most abundant transcripts in androgen-stimulated LNCaP cells by EST count.

**Count**	**Ensembl Gene ID**	**Description**
22377	ENSG00000198899*	ATP synthase a chain (EC 3.6.3.14) (ATPase protein 6).
7595	ENSG00000198916*	No description
3200	ENSG00000148341	SH3 domain GRB2-like protein B2 (Endophilin B2).
2112	ENSG00000198804*	Cytochrome c oxidase subunit 1 (EC 1.9.3.1) (Cytochrome c oxidase polypeptide I).
1678	ENSG00000198886*	NADH-ubiquinone oxidoreductase chain 4 (EC 1.6.5.3) (NADH dehydrogenase subunit 4).
1628	ENSG00000198938*	Cytochrome c oxidase subunit 3 (EC 1.9.3.1) (Cytochrome c oxidase polypeptide III).
1392	ENSG00000186063	No description
1311	ENSG00000198763*	NADH-ubiquinone oxidoreductase chain 2 (EC 1.6.5.3) (NADH dehydrogenase subunit 2).
1201	ENSG00000170421	Keratin, type II cytoskeletal 8 (Cytokeratin 8) (K8) (CK 8).
1088	ENSG00000198744*	No description

* indicates mitochondrial genes

**Table 3:** 25 novel alternative splicing events in androgen-stimulated LNCaP cells

**Ensembl ID**	**Name**	**Description**	**Splice description**
ENST00000248342	eIF3k	Eukaryotic translation initiation factor 3 subunit 12	25 bp deletion of 3' end of exon 1
ENST00000207437	MLEY_HUMAN	Myosin light chain 1, slow-twitch muscle A isoform	64 bp deletion of 5' end of exon 2 (contained in 5' UTR)
ENST00000330964	RPS27L	40S ribosomal protein S27-like protein	Deleteion of retained intron in exon 1 (54 bp of coding sequence)
ENST00000358666	UBL5	Ubiquitin-like protein 5	121 bp deletion of 3' end of exon 1 (contained in 5' UTR)
ENST00000262746	PRDX1	Peroxiredoxin 1	227 bp deletion of 3' end of exon 1 (contained in 5' UTR)
ENST00000297290	BRI3	Brain protein I3	See Figure 3.
ENST00000341480	MED18	Mediator of RNA polymerase II transcription, subunit 18 homolog	Deletion of retained intron in exon 3 (entriely contained in 3' UTR)
ENST00000270799	RPL11	60S ribosomal protein L11	89 bp deletion of 3' end of exon 2 and 103 bp deletion of 5' end of exon 5
ENST00000303553	NDUFA3	NADH-ubiquinone oxidoreductase B9 subunit	Deletion of 45 bp retained intron in exon 4
ENST00000222673	OGDH (MTpc)	2-oxoglutarate dehydrogenase E1 component, mitochondrial precursor	Deletion of 210 bp retained intron (contained in 3' UTR)
ENST00000361643	This gene can be found on Chromosome MT at location 1,673–3,230.		Deletion of 47 bps
ENST00000361390	ROPN1B (MT)	NADH-ubiquinone oxidoreductase chain 1	Deletion of 648 bp
ENST00000302192	Q8WUV6_HUMAN	Podocalyxin-like 2	Deletion of 363 bp retained intron in exon 8
ENST00000361643	This gene can be found on Chromosome MT at location 1,673–3,230.		Deletion of 1268 bps
ENST00000261798	CSNK1A1	Casein kinase I, alpha isoform	Insertion of unknown length between exons 4 and 5
ENST00000339892	This gene can be found on Chromosome 1 at location 234,416,172–234,416,946.		Deletion of 42 bp in 3' UTR
ENST00000322297	OAZ1	Ornithine decarboxylase antizyme	Deletion of last 4 coding bp and 54 bp of 3' UTR
ENST00000224892	LHPP	Phospholysine phosphohistidine inorganic pyrophosphate phosphatase	Deletion of last 105 bp of exon 1, exons 2–6, and first 608 bp of exon 7
ENST00000361381	NU4M_HUMAN (MT)	NADH-ubiquinone oxidoreductase chain 4	Deletion of 152 bp
ENST00000358666	UBL5	Ubiquitin-like protein 5	80 bp deletion of 3' end of exon 1 (contained in 5' UTR)
ENST00000239377	PCMT1	Protein-L-isoaspartate(D-aspartate) O-methyltransferase	47 bp deletion of 3' end of exon 7 (10 bp coding, remainder in 5' UTR)
ENST00000361899	ATP6 (MT)	ATPase protein 6	Deletion of 91 bp
ENST00000291565	PDXK	Pyridoxal kinase	Deletion of 281 bp retained intron (contained in 3' UTR)
ENST00000308964	This gene can be found on Chromosome 19 at location 60,851,213–60,856,333.		42 bp deletion of 3' end of exon 2, exons 3–5, and first 541 bp of exon 6
ENST00000361390	ROPN1B(MT)	NADH-ubiquinone oxidoreductase chain 1	Deletion of 346 bp

**Table 4:** The HQD count, mean and median phred scores and the HQD count

**HQD Type**	**Count**	**Mean**	**Median**	**Count > 400**
**Confirmable**	86	342.4	163	16
**Novel**	1364	270.2	136	175
**Other**	29	233.2	122	4

Counts collected for phred scores > 400 for each of the three HDQ classes: those confirmable by Ensembl, those that occur in positions with no known variations, and those that have incorrect mutations at positions with known variations ("Other").

**Table 5:** Idetification of contamination of 454 EST data

**Species of Origin**	**Number of ESTs**
E. coli	133
Enterococcus sp.	86
Staphylococcus sp.	78
Cloning vector	42
P. marinus (Sea Lamprey)	37
Other	211
**Total**	**587**
